# Supplementary figures and images for: The Rab GTPase activating protein TBC-2 regulates endosomal localization of DAF-16 FOXO and lifespan
Source: PLoS Genet. 2022 Aug 1;18(8):e1010328. doi: 10.1371/journal.pgen.1010328 (PMC9371356; doi:10.1371/journal.pgen.1010328)

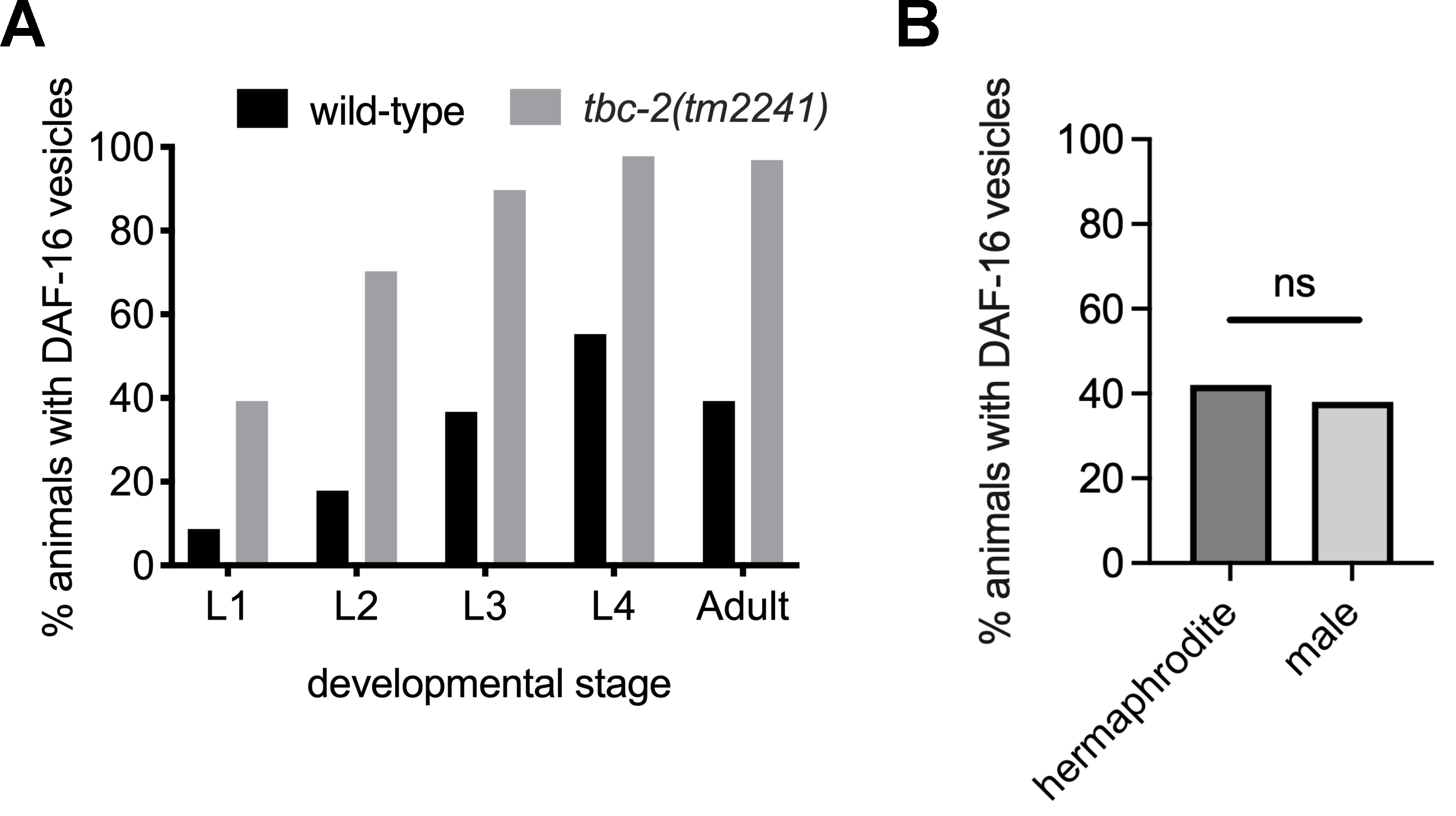

Supplement: S1 Fig — (A) Bar graph of the percent animals with DAF-16a::GFP (zIs356) positive vesicles in the intestinal cells at larval stages L1-L4 and young adults of wild-type and tbc-2(tm2241) animals at 20°C. Fisher’s exact test (graphpad.com) was used to determine that there is a significant increase the number of tbc-2(tm2241) animals with DAF-16a::GFP as compared to wild type at each developmental stage (L1: P<0.05, L2-adult: P<0.0001, n = 23 to 47 animals). (B) Bar graph of the percent zIs356/+ L4/young adult hermaphrodites and males with DAF-16a::GFP positive vesicles. Raw data is available in S1 Data. ns, not significant, n = 47–50 animals. (TIF) [file pgen.1010328.s001.tif]

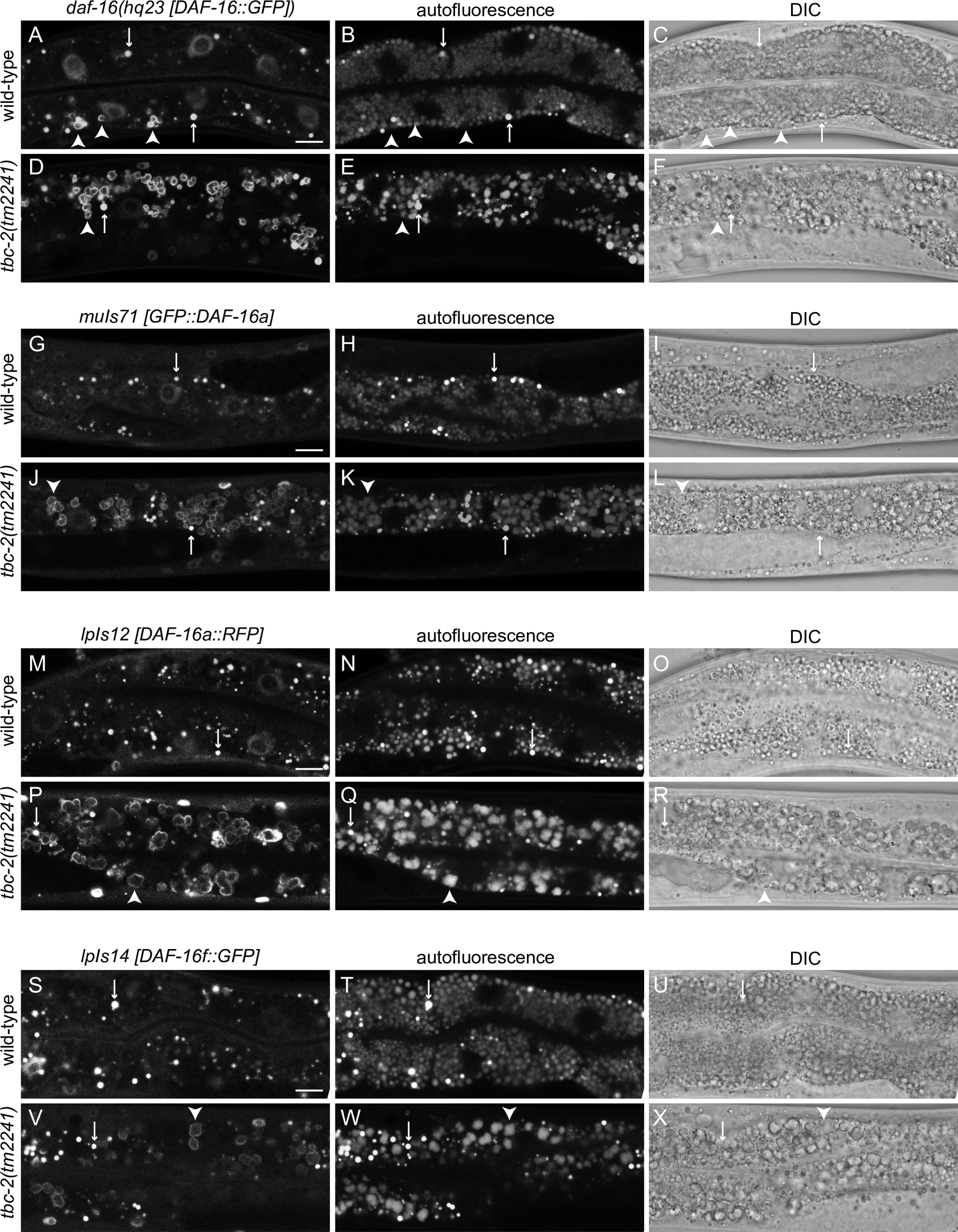

Supplement: S2 Fig — Representative confocal and differential interference contrast (DIC) images of intestinal cells of wild-type (A-C, G-I, M-O and S-U) and tbc-2(tm2241) (D-F, J-L, P-R and V-X) animals expressing DAF-16::GFP daf-16(hq23) (A-F), muIs71 GFP::DAF-16a (G-L), lpIs12 DAF-16a::RFP (M-R) and lpIs14 DAF-16f::GFP (S-X). Endogenously tagged DAF-16::GFP daf-16(hq23) is present on vesicles in both wild-type and tbc-2(tm2241) intestinal cells (A and D arrowheads). Arrows mark bright autofluorescent lysosome-related organelles that bleed through the GFP channel in these lower expressing strains. Vesicular localization of GFP::DAF-16a (G and J), DAF-16a::RFP (M and P) and DAF-16f::GFP (S and V) was only seen in tbc-2(tm2241) animals and not visible in wild-type backgrounds. Scale bars (A, G, M, S), 10μm. (TIF) [file pgen.1010328.s002.tif]

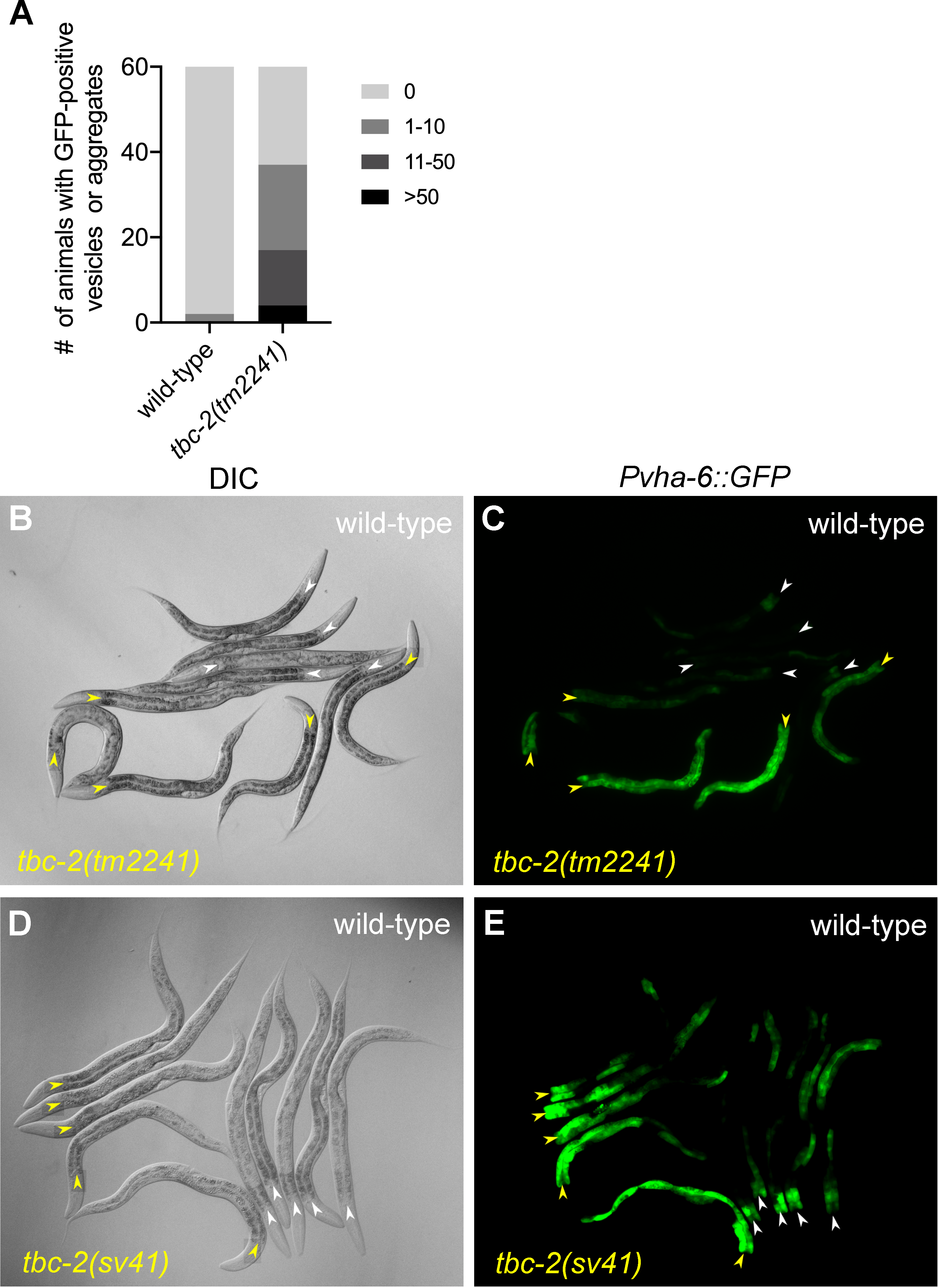

Supplement: S3 Fig — (A) Grouped bar graph quantifying the number of wild-type and tbc-2(tm2241) L4 larvae with 0, 1–10, 11–50 or >50 GFP (vhEx1[Pvha-6::GFP]) positive vesicles or aggregates (aggregates were included in the analyses which are not often seen with DAF-16a::GFP). Raw data is available in S1 Data. (B-E) DIC and epifluorescence images of wild-type and tbc-2(tm2241) (B,C) as well as wild-type and tbc-2(sv41) (D,E) animals expressing GFP under an intestine specific promoter, vhEx1 [Pvha-6::GFP]. White and yellow arrowheads mark the anterior of the intestine of wild-type and tbc-2 mutant animals, respectively. Both tbc-2 mutants have increased GFP expression as compared to wild-type animals. tbc-2 mutants were distinguished from wild-type by the presence of enlarged vesicles using the 100X objective (not shown). (TIF) [file pgen.1010328.s003.tif]

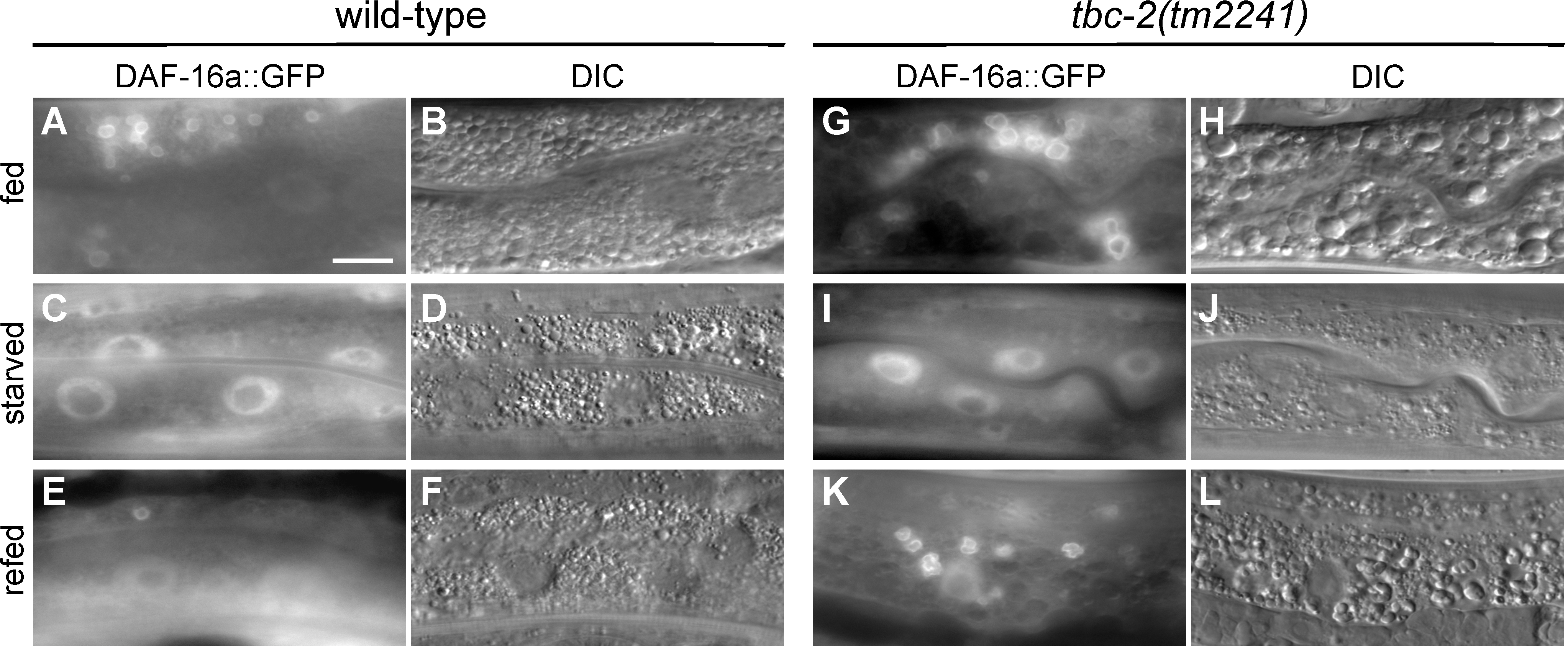

Supplement: S4 Fig — Epifluorescence (A,C,E,G,I,K) and DIC (B,D,F,H,J,L) images of wild-type (A-F) and tbc-2(tm2241) (G-L) animals under fed (A,B,G,H), 4–5 hours of starvation (C,D,I,J) and after 1–2 hours of refeeding (E,F,K,L). Scale bar (A), 10μm. (TIF) [file pgen.1010328.s004.tif]

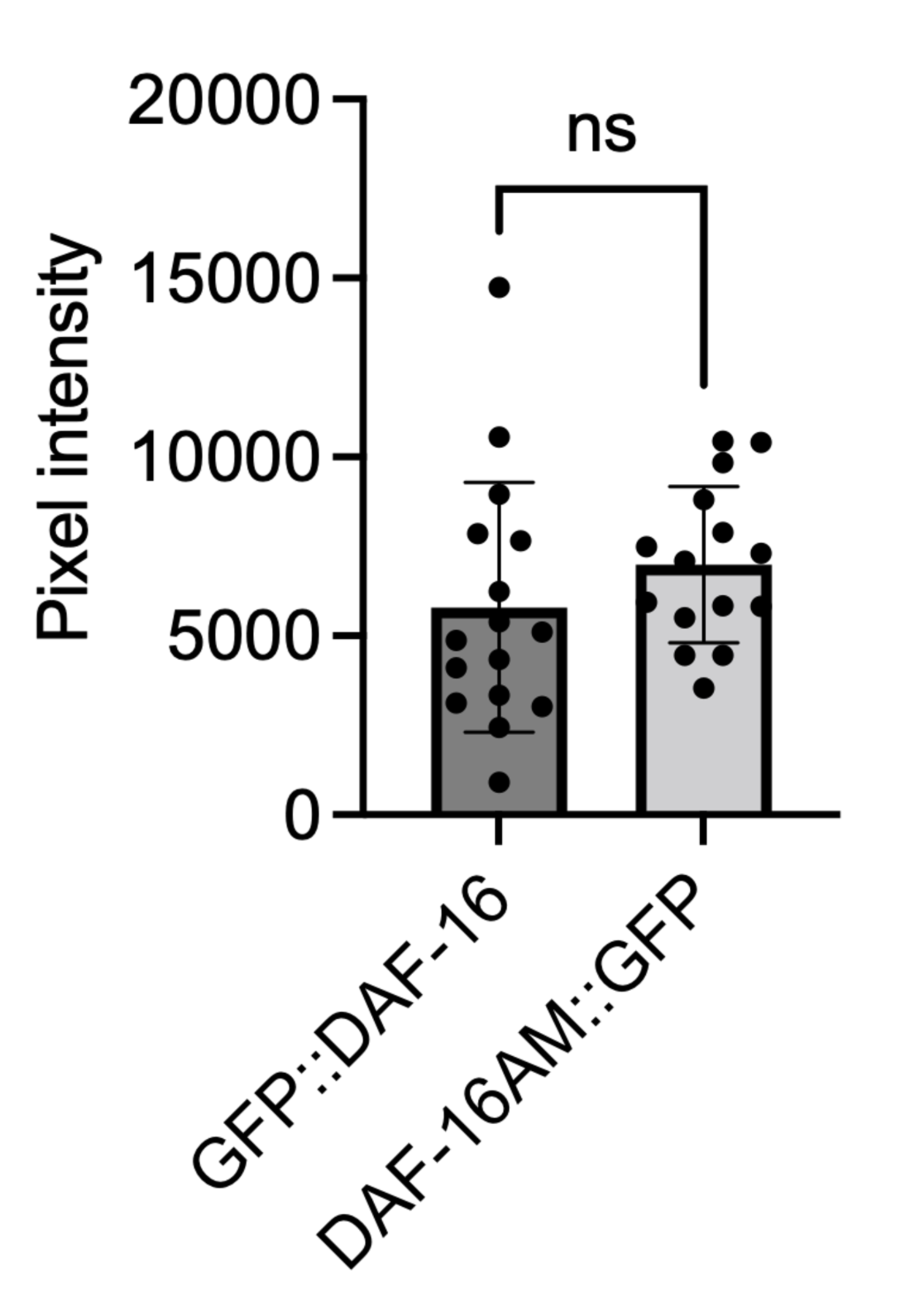

Supplement: S5 Fig — Bar graph depicting the mean pixel intensities of GFP fluorescence in the intestine of QR508 tbc-2(tm2241); muIs71[GFP::DAF-16] and QR697 tbc-2(tm2241); muIs113[DAF-16AM::GFP] animals. Raw data is available in S1 Data. The difference was determined to be not significant (ns) in an unpaired t test. (TIF) [file pgen.1010328.s005.tif]
