# Supplementary material for: The Rab GTPase activating protein TBC-2 regulates endosomal localization of DAF-16 FOXO and lifespan
Source: PLoS Genet. 2022 Aug 1;18(8):e1010328. doi: 10.1371/journal.pgen.1010328 (PMC9371356; doi:10.1371/journal.pgen.1010328)
Supplement: S1 Table — (DOCX) [file pgen.1010328.s006.docx]

**S1 Table. Key resources**

| **Resources Table** | | | | |
| --- | --- | --- | --- | --- |
| **Reagent type (species) or resource** | **Designation** | **Source or Reference** | **Identifier** | **Additional information** |
| Bacterial strain *(E. coli)* | HB101 | Caenorhabditis Genetics Center, CGC |  | Nematode food source |
| Bacterial strain *(E. coli)* | HT115(DE3) | CGC |  | RNAi feeding strain; Used in Figs 3G,3H,4F,4H |
| Genetic reagent | empty vector (ev) L4440 RNAi feeding plasmid | Addgene |  | Used in Figs 3G,3H,4F,4H |
| Genetic reagent | empty vector (EV) RNAi feeding strain | This study |  | HT115(DE3) expressing L4440 RNAi clone, Used in Figs 3G,3H,4F,4H |
| Genetic reagent | RNAi feeding strains *rab-5* (I-4J01), *rab-7* (II-8G13), *akt-1* (V-7I17), *ftt-2* (X-5F07) and *par-5* (IV-6E06) | [1, 2] |  | Used in Figs 3G,3H,4F,4H |
| Nematode Strain (*C. elegans*) | CB1370 | CGC | *daf-2(e1370) III* | Used in Figs 5 and 6 |
| Nematode Strain (*C. elegans*) | CF1407 | CGC | *daf-16(mu86) I; muIs71 [Pdaf-16a::GFP::daf-16a(bKO)) + rol-6(su1006)] X* | Used in S2G-I Fig |
| Nematode Strain (*C. elegans*) | HT1888 | CGC | *daf-16(mgDf50) I;* *unc-119(ed3) III;* *IpIs12[daf-16a::RFP + unc-119(+)]* | Used in S2M-O Fig |
| Nematode Strain (*C. elegans*) | HT1889 | CGC | *daf-16(mgDf50) I; unc-119(ed3) III; lpIs14 [daf-16f::GFP + unc-119(+)]* | Used in S2S-U Fig |
| Nematode Strain (*C. elegans*) | MQD1543 | CGC | *daf-16(hq23[DAF-16::GFP]) I* | Used in S2A-C Fig |
| Nematode Strain (*C. elegans*) | N2 | CGC | wild type | Used in Figs 5 and 6 |
| Nematode Strain (*C. elegans*) | [OH13908](https://cgc.umn.edu/strain/OH13908) | CGC | *daf-16(ot821[daf-16::mKate2::3xFlag]) I* | Used in Fig 2B,2D,2F,2H |
| Nematode Strain (*C. elegans*) | [OH14125](https://cgc.umn.edu/strain/OH14125) | CGC | *daf-16(ot853[daf-16::linker::mNeonGreen::3xFlag::AID]) I* | Used in Fig 2A,2C,2E,2G,2I |
| Nematode Strain (*C. elegans*) | QR15 | CGC | *tbc-2(tm2241) II* | Used in Figs 5 and 6 |
| Nematode Strain (*C. elegans*) | QR150 | This study | *tbc-2(tm2241) II; daf-2(e1370) III* | Used in Figs 5 and 6 |
| Nematode Strain (*C. elegans*) | QR245 | [3] | *unc-119(ed3) III; vhEx1[Plin-31::GFP::rab-7 + Pvha-6::GFP + Cb-unc-119(+)]* | Used in S3 Fig |
| Nematode Strain (*C. elegans*) | QR272 | This study | *tbc-2(tm2241) II;* *zIs356 IV* | Used in Figs 1B,1D,1F,1H,1K, 3G,3H, 4A,4D-F,4H, and S1A Fig, S4A-F Fig |
| Nematode Strain (*C. elegans*) | QR340 | This study | *zIs356 IV; pwIs480[Pvha-6::RFP::rab-5 + Cb unc-119(+)]* | Used in Fig 3A-C |
| Nematode Strain (*C. elegans*) | QR505 | This study | *tbc-2(tm2241) II; lpIs14 [daf-16f::GFP + unc-119(+)]* | Used in S2V-X Fig |
| Nematode Strain (*C. elegans*) | QR508 | This study | *tbc-2(tm2241) II; muIs71 [Pdaf-16a::GFP::daf-16a(bKO)) + rol-6(su1006)] X* | Used in Fig 4I, S5 Fig |
| Nematode Strain (*C. elegans*) | QR646 | This study | *daf-18(e1375) zIs356 IV* | Used in Fig 4D |
| Nematode Strain (*C. elegans*) | QR647 | This study | *daf-18(e1375) zIs356 IV* | Used in Fig 4D |
| Nematode Strain (*C. elegans*) | QR648 | This study | *daf-18(e1375) zIs356 IV* | Used in Fig 4D |
| Nematode Strain (*C. elegans*) | QR651 | This study | *zIs356 IV; pwIs429 [Pvha-6::mCherry::rab-7 + Cb unc-119(+)]* | Used in Fig 3D-3F |
| Nematode Strain (*C. elegans*) | QR655 | This study | *tbc-2(tm2241) II; vhEx1* | Used in S3A-3C Fig |
| Nematode Strain (*C. elegans*) | QR658 | This study | *daf-18(ok480) zIs356 IV* | Used in Fig 4D |
| Nematode Strain (*C. elegans*) | QR659 | This study | *daf-18(ok480) zIs356 IV* | Used in Fig 4D |
| Nematode Strain (*C. elegans*) | QR660 | This study | *daf-18(ok480) zIs356 IV* | Used in Fig 4D |
| Nematode Strain (*C. elegans*) | QR661 | This study | *daf-18(ok480) zIs356 IV* | Used in Fig 4D |
| Nematode Strain (*C. elegans*) | QR662 | This study | *daf-18(ok480) zIs356 IV* | Used in Fig 4D |
| Nematode Strain (*C. elegans*) | QR664 | This study | *tbc-2(tm2241) II; lpIs12 [daf-16a::RFP + unc-119(+)]* | Used in S2P-R Fig |
| Nematode Strain (*C. elegans*) | QR688 | This study | *zIs356 IV; akt-2(ok393) X* | Used in Fig 4E |
| Nematode Strain (*C. elegans*) | QR689 | This study | *zIs356 IV; akt-2(ok393) X* | Used in Fig 4E |
| Nematode Strain (*C. elegans*) | QR697 | This study | *tbc-2(tm2241) II; muIs113 [Pdaf-16::gfp::daf-16AM + rol-6(su1006)]* | Used in Fig 4I, S5 Fig |
| Nematode Strain (*C. elegans*) | QR729 | This study | *daf-16(hq23[DAF-16::GFP]) I; tbc-2(tm2241) II* | Used in S2D-F Fig |
| Nematode Strain (*C. elegans*) | QR779 | This study | *zIs356[Pdaf-16::daf-16a/b(D484V)::GFP + rol-6(su1006)] IV* | TJ356 outcrossed to N2 6; used in Figs 1I, and 4C,4G |
| Nematode Strain (*C. elegans*) | QR807 | This study | *tbc-2(tm2241) II; zIs356 IV* | Derived from QR779; used in Fig 1J |
| Nematode Strain (*C. elegans*) | QR851 | This study | *daf-2(e1370) III;* *zIs356 IV* | Used in Fig 4C |
| Nematode Strain (*C. elegans*) | QR852 | This study | *daf-2(e1370) III;* *zIs356 IV* | Used in Fig 4C |
| Nematode Strain (*C. elegans*) | QR853 | This study | *daf-2(e1370) III;* *zIs356 IV* | Used in Fig 4C |
| Nematode Strain (*C. elegans*) | QR869 | This study | *tbc-2(sv41) II; daf-2(e1370) III* | Used in Fig 5 and 6 |
| Nematode Strain (*C. elegans*) | QR910 | This study | *vhEx1 [Plin-31::GFP::rab-7 + Pvha-6::GFP + Cb-unc-119(+)]* | Used in S2D,E Fig |
| Nematode Strain (*C. elegans*) | QR915 | This study | *tbc-2(sv41) II; vhEx1* | Used in S2D,E Fig |
| Nematode Strain (*C. elegans*) | QR1057 | This study | *daf-16(ot853[daf-16::linker::mNeonGreen::3xFlag::AID]) I; tbc-2(tm2241) II* | Used in Fig 2I |
| Nematode Strain (*C. elegans*) | TJ356 | CGC | *zIs356[Pdaf-16::daf-16a/b(D484V)::GFP + rol-6(su1006)] IV* | Used in Figs 1A,1C,1E,1G, 3G,3H, 4A,4D-F,4H, S1A, & S4A-F |
| Nematode Strain (*C. elegans*) | UP1224 | [4] | *tbc-2(sv41) II* | Used in Figs 5 and 6 |
| Nematode Strain (*C. elegans*) |  | [5] | *daf-16(mu86) I; zIs356 IV; sgk-1(ft15) X* | Used in Fig 4G |
| Nematode Strain (*C. elegans*) |  | [5] | *daf-16(mu86) I; zIs356 IV; sgk-1(ok538) X* | Used in Fig 4G |
| Nematode Strain (*C. elegans*) |  | This study | *zIs356 IV; him-5(e1467) V* | Used in S1B Fig |
| Sequence based reagent | *act-3* qRT-PCR primers | [6] | Forward 5’- TGC GAC ATT GAT ATC CGT AAG G -3’  Reverse 5’- GGT GGT TCC TCC GGA AAG AA -3’ | Used in Fig 6 |
| Sequence based reagent | *sod-3* qRT-PCR primers | [6] | Forward 5’- AAA GGA GCT GAT GGA CAC TAT TAA GC -3’  Reverse 5’- AAG TTA TCC AGG GAA CCG AAG TC -3’ | Used in Fig 6 |
| Sequence based reagent | *dod-3* qRT-PCR primers | [6] | Forward 5’- AAG TGC TCC GAT TGT TAC GC -3’  Reverse 5’- ACA TGA ACA CCG GCT CAT TC -3’ | Used in Fig 6 |
| Sequence based reagent | *mtl-1* qRT-PCR primers | [6] | Forward 5’- ATG GCT TGC AAG TGT GAC TG -3’  Reverse 5’- GCT TCT GCT CTG CAC AAT GA -3’ | Used in Fig 6 |
| Sequence based reagent | *ftn-1* qRT-PCR primers | [6] | Forward 5’- GAG TGG GGA ACT GTC CTT GA -3’  Reverse 5’- CGA ATG TAC CTG CTC TTC CA -3’ | Used in Fig 6 |
| Sequence based reagent | *gpd-2* qRT-PCR primers | [6] | Forward 5’- CTC CAT CGA CTA CAT GGT CTA CTT G -3’  Reverse 5’- AGC TGG GTC TCT TGA GTT GTA GAC -3’ | Used in Fig 6 |
| Sequence based reagent | *icl-1* qRT-PCR primers | [6] | Forward 5’- TGT GAA GCC GAG GAC TAC CT -3’  Reverse 5’- TCT CCG ATC CAA GCT GAT CT -3’ | Used in Fig 6 |

**References**

1. Fraser AG, Kamath RS, Zipperlen P, Martinez-Campos M, Sohrmann M, Ahringer J. Functional genomic analysis of *C. elegans* chromosome I by systematic RNA interference. Nature. 2000;408(6810):325-30.

2. Kamath RS, Fraser AG, Dong Y, Poulin G, Durbin R, Gotta M, et al. Systematic functional analysis of the *Caenorhabditis elegans* genome using RNAi. Nature. 2003;421(6920):231-7.

3. Skorobogata O, Rocheleau CE. RAB-7 antagonizes LET-23 EGFR signaling during vulva development in *Caenorhabditis elegans.* PLoS One. 2012;7(4):e36489.

4. Chotard L, Mishra AK, Sylvain MA, Tuck S, Lambright DG, Rocheleau CE. TBC-2 regulates RAB-5/RAB-7-mediated endosomal trafficking in *Caenorhabditis elegans*. Mol Biol Cell. 2010;21(13):2285-96.

5. Chen AT, Guo C, Dumas KJ, Ashrafi K, Hu PJ. Effects of *Caenorhabditis elegans sgk-1* mutations on lifespan, stress resistance, and DAF-16/FoxO regulation. Aging Cell. 2013;12(5):932-40.

6. Senchuk MM, Dues DJ, Schaar CE, Johnson BK, Madaj ZB, Bowman MJ, et al. Activation of DAF-16/FOXO by reactive oxygen species contributes to longevity in long-lived mitochondrial mutants in *Caenorhabditis elegans*. PLoS Genet. 2018;14(3):e1007268.
